# The role of machine learning in HIV risk prediction

**DOI:** 10.3389/frph.2022.1062387

**Published:** 2022-12-22

**Authors:** Joshua Fieggen, Eli Smith, Lovkesh Arora, Bradley Segal

**Affiliations:** ^1^ School of Public Health and Family Medicine, Faculty of Health Sciences, University of Cape Town, Cape Town, South Africa; ^2^ Phithos Technologies, Johannesburg, South Africa; ^3^ Department of Biomedical Engineering, University of the Witwatersrand, Johannesburg, South Africa

**Keywords:** HIV, machine learning, risk prediction, artificial intelligence, prevention, PrEP

## Abstract

Despite advances in reducing HIV-related mortality, persistently high HIV incidence rates are undermining global efforts to end the epidemic by 2030. The UNAIDS Fast-track targets as well as other preventative strategies, such as pre-exposure prophylaxis, have been identified as priority areas to reduce the ongoing transmission threatening to undermine recent progress. Accurate and granular risk prediction is critical for these campaigns but is often lacking in regions where the burden is highest. Owing to their ability to capture complex interactions between data, machine learning and artificial intelligence algorithms have proven effective at predicting the risk of HIV infection in both high resource and low resource settings. However, interpretability of these algorithms presents a challenge to the understanding and adoption of these algorithms. In this perspectives article, we provide an introduction to machine learning and discuss some of the important considerations when choosing the variables used in model development and when evaluating the performance of different machine learning algorithms, as well as the role emerging tools such as Shapely Additive Explanations may play in helping understand and decompose these models in the context of HIV. Finally, we discuss some of the potential public health and clinical use cases for such decomposed risk assessment models in directing testing and preventative interventions including pre-exposure prophylaxis, as well as highlight the potential integration synergies with algorithms that predict the risk of sexually transmitted infections and tuberculosis.

## Introduction

Since the start of the HIV epidemic, the virus has infect an estimated 76 million people worldwide, roughly 33 million of whom have died (1). While there has been a roughly 60% reduction in estimated AIDS-related annual deaths this progress has not been reflected in HIV incidence with only a 17% decrease in HIV incidence across a similar period leading to a significant rise in the number of people living with HIV (1–3). In recognition of the limited successes in reducing HIV infection incidence globally, the UNAIDS “Fast-track” targets of 95–95–95 have become accepted as foundational for accelerating HIV incidence reductions to achieve the goal of ending the HIV epidemic by 2030 (4). The updated targets seek to have 95% of people living with HIV know their status (diagnosis), 95% of those diagnosed on antiretroviral therapy (ART), and 95% of those on ART virally suppressed by 2030 (4). Fundamental to achieving this goal in Sub-Saharan Africa is comprehensive diagnosis including populations unaware that they are at high risk of having HIV (5). However, a major challenge to this is that the relative importance of different at-risk and missed groups varies significantly both between and within different countries.

Beyond the Fast-track targets other preventative strategies remain vital to the global efforts to end the HIV epidemic, with Pre-Exposure Prophylaxis (PrEP), behaviour change communication, and early ART as prevention considered to be the three most effective strategies for preventing HIV transmission (6). When taken correctly, both PrEP and ART as prevention has been shown to be up to 100% effective in preventing HIV transmission (7–10). Critical to directing both HIV testing campaigns (required to meet the first goal of 95–95–95) as well as PrEP prescription and other targeted preventative strategies is a capacity for granular HIV risk estimation. This unmet need coupled with the initial successes of more traditional modelling techniques in delineating HIV risk (11), has led to a growing interest in the role machine learning (ML) and artificial intelligence (AI) could play in helping quantify individual risk of HIV infection. To this end, various ML models and AI algorithms have been developed using diverse datasets from both data-rich high income settings and more data-sparse low-to-middle income countries (LMICs) (12–19). In this perspective article we seek to describe the benefits and limitations to using ML for HIV risk prediction as well as discuss some of the potential future use cases of ML-guided HIV risk prediction algorithms in both meeting the UNAIDS targets as well as guiding the roll-out of other preventative strategies such as PrEP.

## Machine learning for HIV risk prediction

Machine learning (ML) can be described as a collection of scientific techniques that focus on how computers learn relationships between data (20, 21). The automated pattern recognition of ML has found growing utility in medical statistics owing to the increasing size and complexity of medical data (22). ML can be classified into supervised or unsupervised learning by whether the algorithm is trained on labelled data or the algorithm self-defines the data structure from unlabelled data (20, 23, 24). Supervised learning can then be further subclassified into classification and regression algorithms based on whether the outcome being predicted is a categorical or continuous variable respectively (24). Common examples of classification problems include email spam filters (25), movie or online shopping recommendations (26), differentiating malignant and benign skin lesions (27), modelling the risk of ICU admission (28), and chest radiograph pneumonia detection (29).

The output of a classification algorithm is typically interpreted as a probability which is then binarized by means of a threshold that can be altered to increase either the sensitivity or specificity based on the model's clinical requirements (30). This makes classification models particularly useful in risk prediction. Given the persistent global burden of infectious diseases, there has been growing interest in the use of ML in risk prediction in this field (31, 32). Within HIV specifically there has been substantial attempt to try and identify individuals at high risk of infection. Initially people were classified using single risk factors, such as sero-discordant spouses (12). Subsequent approaches have largely focused on risk scores calculated *via* traditional clinical prediction tools based on regression modelling (33), with different models attempting to quantify risk of HIV seroconversion among different risk groups including men-who-have-sex-with-men (MSM), women, and sero-discordant couples (11, 34–38). Most recently, various authors have used ML approaches to attempt to quantify the complex relationships between risk factors that contribute to HIV risk (12–19). Balzer et al. directly evaluated these three approaches by comparing traditional risk factors, a risk score estimated by logistical regression, and an ML model estimated using the Super Learner algorithm and showed that ML significantly improved both the efficiency and sensitivity in identifying HIV seroconversions (12).

## ML: model development and evaluation

### Feature selection and model building

The predictor variables used in a ML model are called features. While the ability to handle higher numbers of features and learn the complex associations between them is one of the inherent advantages of ML, in general fewer features reduces the risks of model overfitting and leads to improved generalisability of the algorithm (39). Model overfitting is where a model's predictions are too finely tuned to the statistical noise or spurious statistical correlations in the dataset used to build the model and leads to significant limitations with generalising the model's output to new data (40). For this reason, parsimonious inclusion of features is important. Some supervised learning algorithms inherently only select the most predictive features, while for other algorithms this process needs to be made explicit (41). In addition to careful feature selection and design, another fundamental component of ML model building that helps prevent overfitting is the random splitting of the initial dataset into training, validation, and test datasets (24). Different models are then developed (trained) and compared using the validation dataset with the final model applied to the features of the holdout test dataset to evaluate the model's performance. This explicit separation of data attempts to select for models that have extracted useful features that at a minimum generalise across unseen subsets of the dataset. Variants of this such as a bootstrapping or cross validation exist with the overall gold standard being the use of an external dataset for testing (42).

Within HIV, exact risk factors for transmission vary significantly by population but are typically behavioural and socio-demographic in nature (43). This has led to such factors featuring prominently in ML algorithms attempting to estimate HIV risk (19). The propensity of these features to have complex and poorly understood interactions has given ML-based approaches a distinct advantage in adjusting and quantifying aggregate infection risk but simultaneously introduces particular risk of overfitting. In addition, a significant challenge with modelling HIV risk is that incidence varies significantly by population impacting the prior or baseline probability of infection. A possible way to manage this challenges across difference populations is to include geography as a feature either as a ZIP/postal code (13) or as a longitude, latitude, and altitude (44). Finally, ML is increasingly used in combination with other AI techniques such natural language processing to assist with extracting important features from the narrative text of electronic health records to further enhance an automated process of HIV risk prediction (17).

### Performance metrics

**Figure 1:**
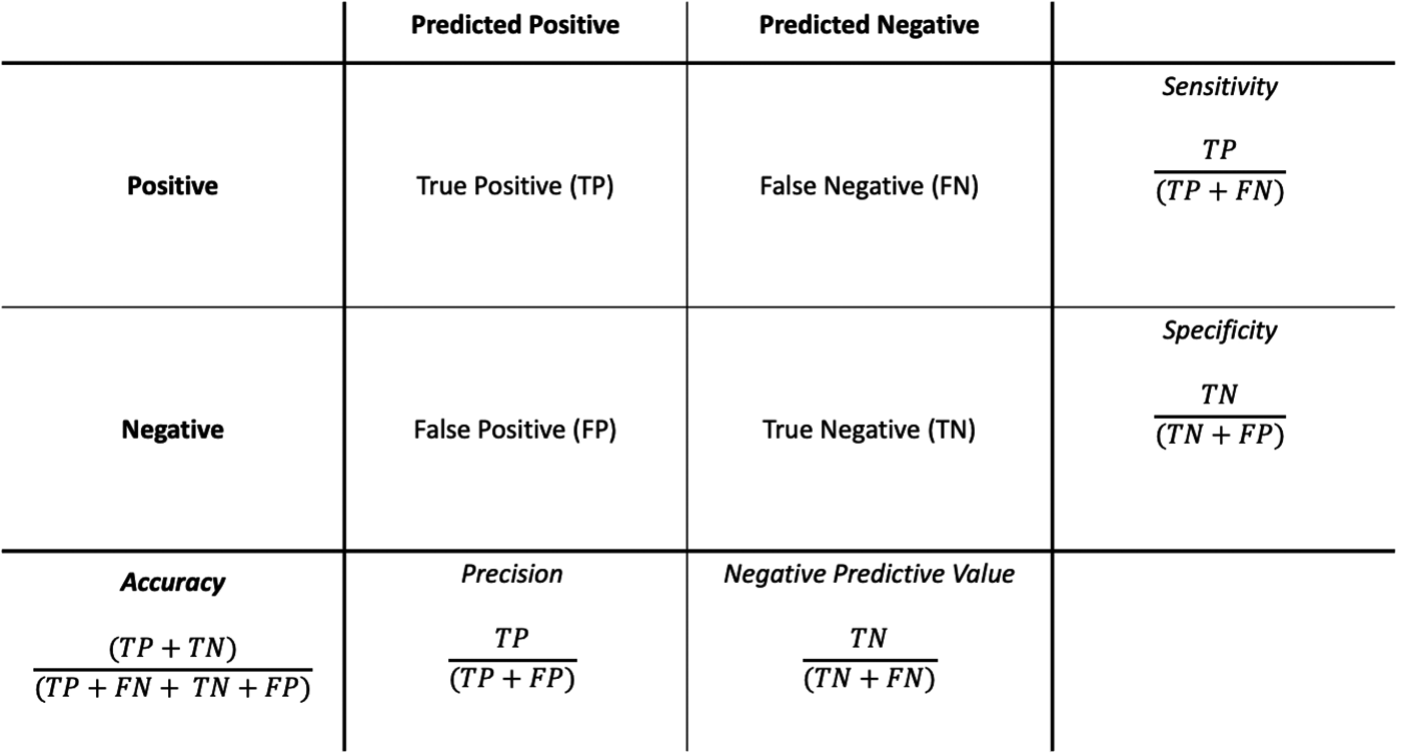
Performance metrics calculated from a confusion matrix.

In contrast to traditional statistics where the primary purpose is inference, in classification ML the primary focus is on accurate prediction (24). Given that prediction is the primary concern, the confusion matrix (Figure 1) is a useful method to assess the performance of a given model. To generate a confusion matrix, the model is applied to the features of the holdout test dataset and the predicted outcomes (at a set probability threshold) are compared to the actual outcomes seen in the dataset. From there the performance metrics of sensitivity, specificity, accuracy, precision, and negative predictive value are calculated. However, one of the challenges with the confusion matrix is the fact the predictions are made at a particular threshold probability and thus it is difficult to assess what would happen at different probability cut-offs. In this respect receiver operator curves (ROCs), and particularly the area under the ROC (AUROC), provides a useful way of visualising and describing the trade-off between sensitivity and specificity in a model at all probability thresholds and is considered among the gold standard measures of ML model performance when applied to clinical risk prediction (45). The AUROC is especially relevant to healthcare applications as the results are not dependent on the relative prevalence of the outcome.

### Understanding the model

ML's advantage in predictive performance often comes at the expense of the more typical research goal of interpretability. A common heuristic in estimating this trade-off is in the number of parameters a model utilises to make predictions (46). A simple logistic regression model has a single parameter per predictive feature whereas large-scale modern deep neural networks may have several billion (47). This presents a clear challenge to utilising ML models in practice as it becomes difficult to trust predictions that are based off unknown combinations of features, especially with concerns that models may automatically learn specific biases inherent to the dataset (48–50). Interpretable ML is the domain interested in combining these two paradigms by providing techniques that enable explanations to be extracted from models several orders of magnitude more complex than is typically feasible (51).

A method that has gained substantial popularity in recent years for this task is the Shapley Additive exPlanations (SHAP) framework (52). These tools utilise an approach rooted in game theory to provide so called ‘SHAP values’. These values indicate the influence each predictive feature exerted on the final model prediction and can be used to gain substantial insight into the most discriminative features a model may utilise. In addition, this assists in model trust by enabling an individual to sanity check the model's feature attribution in making a final determination. This decomposition allows for model utilisation beyond simple prediction, and can be employed to, for example, provide separate estimates of modifiable and non-modifiable risk factors despite the use of a more complex model.

**Figure 2:**
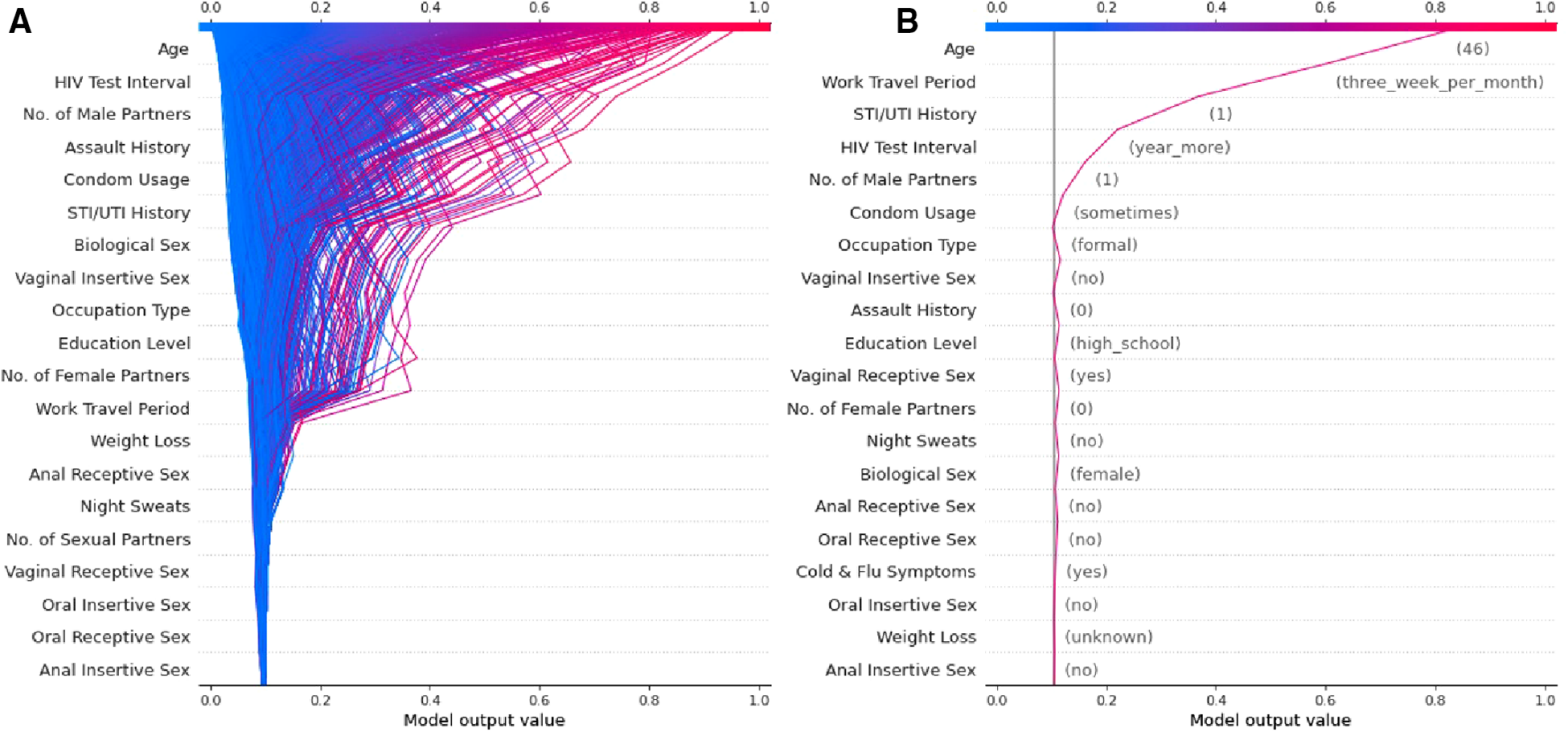
Examples of a SHAP-based feature explanation for a ML model showing (A) overall model risk output for all individuals in the test dataset and (B) an example individual at high risk for contracting HIV.

The authors have developed a ML model using socio-demographic and behavioural data collected prospectively with a digital survey as described in the published protocol (53). The cohort is described in detail in a manuscript currently under review and Figure 2 is a sub-analysis of this data presented as a visual explanation of the potential utility of SHAP-based metrics in decomposing HIV risk at both an aggregate (Figure 2A) and individual (Figure 2B) level. Given that the social and behavioural risk factors for HIV vary by context (43), the relative predictive value of these features seen in Figure 2 is specific to this cohort and likely varies across cultures and regions. This underscores the importance of local validation and fine-tuning of any risk prediction algorithm that utilises socio-behavioural features prior to deployment emphasises the importance of model decomposition in understanding the contributors to risk in a given population.

The main limitation of SHAP-based metrics is that while they provide explanations of how a model reached a particular prediction, they do not quantify how accurate that prediction is (52, 54). Multiple methods exist that attempt to determine the relationship between the inclusion of a variable and overall model performance. Permutation Importance (PIMP) is one such tool that attempts to provide a structured approach to determine variable importance (54). This method randomly shuffles one column of the dataset at a time for several thousand iterations, one set with the outcome preserved and another with the outcome also shuffled. This provides two distributions the overlap of which provides a measure of significance and scale to which a variable improves a model. The main challenges with this methodology are that it can be computationally intensive to run enough replications and that certain variables may be highly correlated and may need to be shuffled together to gain an accurate estimation of importance (54).

## Discussion

### Modifying the public health response: community and individual orientated care

A major limitation to the use of ML models in HIV risk prediction thus far has been the limited interpretability of these models beyond their predictive capacity. However, we argue that tools such as PIMP and particularly SHAP allow these models to have clinical implications beyond simple prediction. Specifically, these tools allow for the decomposition of the features that make up “risk” at both an aggregate level (Figure 2A) and an individual level (Figure 2B). We believe this can translate into clinical practice by facilitating more efficient and targeted use of the interventions currently available.

If a model is appropriately contextualised and locally validated, a feature decomposition such as that presented in Figure 2A should provide an overview of the most important contributors to risk in a given community. In this example, age, duration since last HIV test, and the number of male sexual partners appears to convey the largest risk component. These features can then be considered in terms of modifiable risk (e.g., low levels of condom usage) or non-modifiable risk (e.g., high rates of work travel) and the public health response tailored towards either behaviour change communication or PrEP as guided by a given population's overall risk distribution. Similarly, by identifying specific risks at the individual level (Figure 2B), one is able to offer directed counselling and personalised interventions for risk factors that are most impacting the individual's chance of contracting HIV. For example, the major contributors to risk for the example individual in Figure 2B are non-modifiable and thus they may well be a good candidate for PrEP and counselling for this intervention can be directed by the risk profile generated. By identifying these factors, public health interventions could be targeted at specific issues rather than attempting to solve a heterogenous problem with blanket solutions that are not necessarily applicable to specific individuals or communities.

### Utility in PrEP initiation

PrEP is widely regarded as one of the most effective strategy in the prevention of HIV transmission (55–57) and has been shown to be a cost-effective method to address the HIV epidemic (58). The recent advent of an injectable PrEP preparation containing cabotegravir heralds much excitement as the drug persists for long periods in those exposed allowing long intervals between dosing (56) which is required only every second month. The ease of administration this enables promises to alleviate some of the adherence issues faced in PrEP strategies (56). The agent has recently been approved by the Federal Drug Administration and is currently under review by various local agencies including in some LMICs, providing an opportunity to renew efforts to promote large-scale global uptake of PrEP.

Much of the current discussion around PrEP strategies centres around the issue of to whom PrEP should be offered (14, 16, 55). Identification of individuals at risk forms the basis of this discussion. Thus far strategies have directed PrEP administration at particular population groups such as MSM or particular geographical regions known to have a high prevalence of HIV (14), however there is a need to better identify candidates for PrEP in order to optimize its benefit (16). Methods in this area have aimed to identify individuals that would glean the greatest benefit from PrEP administration by identifying individuals at the greatest risk of HIV acquisition or seroconversion (14). Recognition of various individual level data as conferring risk for seroconversion has been the topic of much literature. These factors include non-modifiable factors such as age, sex, sexual-orientation and behaviour, as well as modifiable factors such as number of sexual partners or condom use (14, 16). Combinations of various factors of this type have been used to identify the most at-risk individuals and therefore those that would benefit most from PrEP. The complex matrix of data points that arises from analysis of this data is not always captured by simple calculations of risk. As such, there is significant benefit to ML as a method to augment the use of such data (14, 16). These strategies allow the capturing of the intricate interaction between factors and better identifies individuals at risk of contracting HIV and seroconverting. By using these methodologies, the efficient use of PrEP is increased as its administration is targeted at individuals with a greater likelihood of contracting HIV.

### Future uses of ML in HIV associated conditions

Given the important interactions between the risk factors for sexually transmitted infections (STIs) and HIV and ML's strength in this area, it is logical to build an integrated tool that predicts the risk of both conditions. Xu et al. (2022) have recently built such a tool with a web-based interface that delivered reasonable predictive performance for HIV (AUROC = 0.72), syphilis (AUROC = 0.75), gonorrhoea (AUROC = 0.73), and chlamydia (AUROC = 0.67) (18). Given the biologically-based increased risk of HIV infection conferred by STIs (59), as well as the persistent use of syndromic management in treating STIs in many LMICs (60), algorithms that incorporate both conditions likely have significant potential synergies and utility in LMIC settings. In addition, tuberculosis (TB) is perhaps the most importance HIV-associated disease as it is estimated be responsible for around a third of deaths among people living with HIV (61). ML has been shown to be effective in assisting with both the screening and diagnosis of TB as well as predicting the risk of TB drug resistance (62). However, to the best of our knowledge ML-based TB and HIV risk assessment models have not yet been integrated into a single tool. While the combination of TB and HIV prediction algorithms offers less potential predictive synergy there is valuable overlap in possible clinical utility.

## Conclusion

As authors, we believe ML as applied to HIV risk prediction has the potential to make a significant contribution towards ending the HIV epidemic. Specifically, we see it as a critical tool in directing testing, behaviour change communication, and PrEP towards individuals and communities at high risk of infection in a resource efficient manner. Yet, while these models have been shown to be scientifically valid there remain significant barriers to them having a tangible impact. The most important of these challenges include establishing the tools for the collection of socio-demographic and behavioural data, the appropriate contextualisation and local validation of models, and the successful integration of such systems into routine HIV prevention services, particularly in resource constrained LMIC settings.

## Data Availability

The original contributions presented in the study are included in the article/Supplementary Materials, further inquiries can be directed to the corresponding author/s.
